# The Role of Syk/CARD9-Coupled C-Type Lectin Receptors in Immunity to *Mycobacterium tuberculosis* Infections

**DOI:** 10.1155/2010/567571

**Published:** 2011-01-09

**Authors:** Mohlopheni Jackson Marakalala, Lisa M. Graham, Gordon D. Brown

**Affiliations:** ^1^Institute of Infectious Diseases and Molecular Medicine, Division of Immunology, CLS, University of Cape Town, Cape Town 7925, South Africa; ^2^Aberdeen Fungal Group, Section of Immunology and Infection, Division of Applied Medicine, Institute of Medical Sciences, University of Aberdeen, Aberdeen AB25 2ZD, UK

## Abstract

There is increasing interest in understanding the mechanisms underlying the interactions that occur between *Mycobacterium tuberculosis* and host innate immune cells. These cells express pattern recognition receptors (PRRs) which recognise mycobacterial pathogen-associated molecular patterns (PAMPs) and which can influence the host immune response to the infection. Although many of the PRRs appear to be redundant in the control of *M. tuberculosis* infection *in vivo*, recent discoveries have revealed a key, nonredundant, role of the Syk/CARD9 signalling pathway in antimycobacterial immunity. Here we review these discoveries, as well as recent data investigating the role of the Syk/CARD9-coupled PRRs that have been implicated in mycobacterial recognition, including Dectin-1 and Mincle.

## 1. Introduction

Despite over a century of research, tuberculosis (TB) remains the deadliest bacterial infection causing about 1.6 million deaths per year worldwide [1]. In most cases of TB infection, the immune response is successful in containing *Mycobacterium tuberculosis* (MTB), which then remains in a dormant, nonreplicative state, termed latency. This clinical latency often extends for the lifetime of the individual but reactivation of the infection can occur due to perturbations of the immune system [2]. An estimated one-third of the world's population are latently infected with MTB, and about 10% of these people are likely to become sick with active TB in their lifetime. The emergence of new resistant strains as well as coinfection with HIV also provides more challenges in the treatment of TB, making it a global problem that needs urgent attention [1]. Thus development of new antitubercular drugs and a more effective vaccine is a priority; both of which depend, in large part, on obtaining a better understanding of interaction between the host and this pathogen.

The immune system of humans and other mammals is comprised of two interrelated components, the innate and adaptive response, both of which are required for the resolution of the infection. The innate immune system, in particular, consists of phagocytes such as macrophages and dendritic cells which are involved in the initial capture of the bacteria, as well as the presentation of microbial antigens. Serving as first line of defence, these innate cells express a variety of evolutionarily conserved pattern recognition receptors (PRRs), which are involved in the recognition of pathogen-associated molecular patterns (PAMPs) [3]. 

One of the first events after the inhaled mycobacteria have reached the alveolar space is its recognition by alveolar macrophage PRRs. The role of PRRs, such as the toll-like receptors (TLRs) and NOD-like receptors (NLRs), in antimycobacterial immunity has been a focus of considerable interest [4, 5]. TLR-2, TLR-4, for example, were shown to play a role in the long-term control of *M. tuberculosis *[4]. Furthermore, TLR-9 was reported to be important in the induction of protective responses, and in cooperation with TLR-2, to mediate resistance to MTB infection [6]. Studies using MyD88-deficient mice also demonstrated a role of this downstream adaptor in the acute control of MTB infection [7]. However, recent data have shown that MyD88 and TLR-2, -4, and -9 are dispensable for the induction of T cell-mediated adaptive immunity to MTB infection [8, 9]. TLR-2, -4, and -9-deficient mice were also not impaired in their macrophage effector mechanisms and displayed normal control of MTB replication [8]. Thus, the TLRs, even in combination, appear to be redundant for the control of MTB infection.

Nucleotide-binding oligomerization domain proteins (NODs) are modular cytoplasmic proteins that recognise components of peptidoglycan [4]. NOD2 is the widest studied NLR in the MTB infections and has been shown to synergize with TLR-2 for the production of proinflammatory cytokines in response to MTB. Gandotra et al. have reported that NOD2-deficient macrophages and DCs were impaired in the production of proinflammatory cytokines in response to live MTB. However this NLR seems to be dispensable in the *in vivo* control of the infection, as NOD2-deficient and the wild-type control mice show similar levels of susceptibility when infected with virulent MTB [10]. More work is needed to understand the role of other NLRs in antimycobacterial infections. 

Other PRRs have also been implicated in the immune response to *M. tuberculosis*, including the mannose receptor (MR), complement receptor 3 (CR3), dendritic cell-specific intercellular adhesion molecule 3 (ICAM-3)-grabbing nonintegrin (DC-SIGN), class A scavenger receptor, mannose binding lectin (MBL), and surfactant protein A (Sp-A) [11, 12]. Nonetheless, each of these receptors also seem to be functionally redundant, as it has been shown that single deficiencies in PRRs such as Scavenger receptor A, CD36, MARCO, mannose receptor, and SIGNR1 do not impair the control of *M. tuberculosis in vivo *[13]. Thus, we and others have speculated that mycobacteria are likely to simultaneously engage multiple receptors and that deficiency of one receptor can be compensated by another [5, 13]. However, recent discoveries suggest that the Syk/CARD9 pathway plays a nonredundant role in antimycobacterial immunity, and two C-type lectin receptors (CLRs) recognising mycobacteria that utilise this pathway have been identified. Here we review our current understanding of the Syk/CARD9 pathway, as well as the two CLRs (macrophage-inducible C-type lectin (Mincle) and Dectin-1), with particular emphasis on their roles in antimycobacterial immunity.

## 2. CARD9 (Caspase Recruitment Domain-Containing Protein 9): An Adaptor Molecule Involved in ITAM-Coupled Receptor Signalling

CARD9 is an adaptor molecule that contains N-terminal caspase recruitment domain (CARD) and a C-terminal coiled-coil region that is important for protein oligomerization [14]. Structurally, this adaptor is related to CARMA (CARD MAGUK) family of proteins, which include CARMA1 (CARD11), CARMA2 (CARD10), and CARMA3 (CARD13) [15]. Originally discovered through a database search of CARD-containing proteins, CARD9 is expressed in various tissues including the lung, liver, spleen, placenta, peripheral blood leukocytes, and bone marrow. CARD9 is not expressed in lymph nodes, T cells, or B cells, but is abundantly expressed in innate myeloid cells including dendritic cells and macrophages [14, 16]. 

This adaptor protein operates downstream of immunoreceptor tyrosine-based activation motif-(ITAM-) associated PRRs and cooperates with B cell lymphoma 10 (Bcl10) and the paracaspase MALT1 to form a trimolecular complex that transduces signalling to the canonical NF*κ*B pathway [15]. CARD9 was initially identified as a key transducer of Dectin-1 signalling [17], but more recently this signalling pathway has also been shown to be important for myeloid cell activation via DAP12- and FcR*γ*-associated receptors [18]. 

Several murine studies have demonstrated that CARD9 plays an essential role in protection against fungal and bacterial pathogens, including *C. albicans* and *Listeria monocytogenes *[16, 17]. A recent study by Glocker et al. has described a CARD9 mutation in humans that is associated with an increased susceptibility to chronic mucocutaneous candidiasis [19]. Interestingly, these patients had significantly reduced numbers of Th17 cells [19], and murine studies have shown that signalling through the CARD9 pathway, from receptors including Dectin-1 (see below) and Dectin-2, is important for the development of these types of adaptive responses [20–22]. Indeed, LeibundGut-Landmann et al. have shown that CARD9 is indispensable for the induction of Th17 responses in mice infected with *C. albicans *[20]. Poeck et al. have recently shown that RIG-I engages CARD9-Bcl10 signalling pathway for the production of IL-1*β* in response to RNA viruses [23]. Thus CARD9 is a central molecule that transduces signals from multiple PRRs to induce immunity to various pathogens.

## 3. CARD9 and Antimycobacterial Immunity

The essential role of CARD9 control of pulmonary tuberculosis, at least in mice, was recently demonstrated by Dorhoi et al. [24]. These authors found that *Card9^−/−^* mice succumbed rapidly to aerosol infection with *M. tuberculosis* H37Rv, and, compared with their littermate controls, the CARD9-deficient mice had significantly higher pulmonary bacillary burdens and more profound tissue damage, characterized by acute pneumonia with accelerated accumulation of inflammatory cells. There was also evidence of secondary necrosis in the *Card9^−/−^* lung tissue, suggesting a correlation between cell death in lungs of these mice and their susceptibility to tuberculosis infection [24]. 

To investigate the effect of CARD9 deficiency in the activation of antigen presenting cells (APCs), Dorhoi et al. infected bone-marrow-derived macrophages (BMMø) with MTB H37Rv and analyzed various innate responses. Both the WT and *Card9^−/−^* BMMø produced similar levels of nitric oxide (NO), and the adaptor deficiency did not have an effect on internalization or killing of MTB after IFN-*γ* activation. The authors also demonstrated that the clearance of apoptotic cells was also not impaired in *Card9^−/−^* BMMø. Furthermore, apoptotic cell death occurred at similar frequency in both the WT and *Card9^−/−^* cells [24]. However, infected *Card9^−/−^* BMMø had significant reductions in the synthesis of proinflammatory cytokines, TNF, IL-1*β*, and IL-6, and produced less IL-12 and CCL5, when compared to the WT cells. The MTB-infected *Card9^−/−^* BMDCs were also impaired in their ability to synthesise TNF and IL-1*β*. These data indicated that CARD9 is critical for activation of innate immune cells following the recognition of MTB [24]. 

To characterize the PRRs involved in APC activation, Dorhoi et al. stimulated BMMø and DCs with MTB-derived PAMPs, including manLam, peptidoglycan (PG), mycolyl-arabinogalactan peptidoglycan (mAGP), and mycobacterial cord factor. The stimulation with manLam and cord factor led to cellular activation, suggesting possible involvement of MR and Mincle [24, 25] (see below). The stimulation of wild-type BMMø and DCs with mAGP triggered the release of TNF and IL-10, which were abrogated in the CARD9-deficient cells. These results suggested the involvement of nucleotide-binding oligomerization domain protein 2 (NOD2) in the CARD9-mediated innate response to MTB [24], as mAGP-induced cytokine production has been previously shown to partially depend on this receptor [10]. Furthermore, the authors showed that the treatment of wild-type BMMø with laminarin (a *β*-glucan antagonist of Dectin-1) and piceatannol (a Syk kinase inhibitor) specifically blocked TNF and IL-6 production, suggesting that signalling through the Dectin-1/Syk pathway was also important in the induction of MTB-triggered proinflammatory responses [24]. Thus, the authors speculated that during MTB recognition CARD9 is likely to converge signals from a number of PRRs to mediate innate responses to this pathogen [24]. 

To characterize the role of CARD9 in adaptive cell responses, Dorhoi et al. measured various T cell populations and the recruitment of leukocytes to the lungs following infection with MTB. At day 14 postinfection, there were no differences between WT and *Card9^−/−^* mice in the recruitment of T cell subpopulations to the lung. Also, the ratio of CD4^+^/CD8^+^ cells in the wild-type and the knockout mice were similar at both days 14 and 28 after infection. Furthermore, there were similar frequencies of CD8^+^ T cells and FoxP3^+^ T lymphocytes in both groups of mice [24]. Thus, CARD9 deficiency does not affect T cell responses in MTB infection. In addition to T cells, the authors showed that there was no difference in the frequency of lung macrophages, DCs, as well as the expression of CD86 (an APC-expressed maturation marker) [24]. 

There was, however, a substantial accumulation of neutrophils in the lungs of CARD9-deficient mice compared to the wild-type control mice. The accelerated recruitment of the polymorphonuclear neutrophils (PMNs) to the lung corresponded to the disease progression, implicating these cells in the susceptibility of *Card9^−/−^* mice to MTB infection [24]. In an endeavour to determine what triggered the neutrophilic inflammation during the infection, the authors measured chemokine and cytokine levels in serum and lung homogenates. Intriguingly, MCP-1, KC, and G-CSF were significantly higher in the *Card9^−/−^* mice. In addition, elevated concentrations of granulocyte-derived myeloperoxidase (MPO) were detected in sera of these mice compared to the wild type control animals. Thus, the authors conclude that the systemic inflammatory disease that leads to tissue damage emanates from G-CSF-mediated augmented granulocyte differentiation and the KC-driven accelerated recruitment of the neutrophils to the lung [24]. Rescue experiments performed by either neutralizing G-CSF or depleting PMN with anti-Gr-1 monoclonal antibody resulted in the reduction of lung inflammation and prolonged survival. Furthermore, the authors also demonstrated that the CARD9-deficient PMNs lost their capacity to produce IL-10 upon MTB challenge and thus failed to fine-tune the level of inflammation in the lung [24]. Collectively, the data by Dorhoi et al. demonstrates an essential role of CARD9 signalling in the control of MTB infection [24]. 

In another recent study, Werninghaus et al. reported that the mycobacterial PAMP, trehalose-6,6-dimycolate (TDM, cord factor), and its synthetic analogue trehalose-6,6-dibehenate (TDB) activate antigen presenting cells, macrophages, and DCs, via the Syk-CARD9 pathway to induce innate responses such as NO production and the release of cytokines such as TNF, IL-1*β*, and IL-6 [25]. TDM was first identified in 1950 by Bloch as a glycolipid that was responsible for cording in tubercle bacilli [26], and six decades later this glycolipid remains one of the widely studied components of mycobacterial cell membrane mainly due to its ability to activate the innate immune system in mammals [27]. Werninghaus et al. showed *in vivo* that these glycolipids could be used as adjuvants to drive combined Th1 and Th17 T cell responses to an MTB subunit vaccine in a CARD9-dependent manner [25]. These responses were also FcR*γ*-dependent and subsequently identified to be mediated through Mincle (see below).

## 4. Dectin-1 Structure, Expression, and Signalling

Dectin-1 is a glycosylated type II transmembrane receptor possessing a single extracellular C-type lectin-like domain (CTLD) and a cytoplasmic signalling domain which is involved in cellular activation [28, 29]. This receptor was first discovered through subtractive cDNA cloning from murine dendritic cells (DCs) [30] and later identified and characterized in humans [31, 32]. In both of these organisms, alternative splicing of Dectin-1 results in two major isoforms and a number of minor isoforms, with the major ones differing in the presence or absence of the stalk region [31, 33]. Dectin-1 is predominantly expressed on myeloid cells, including dendritic cells, monocytes/macrophages, and neutrophils, as well as a subset of T cells [34]. This PRR has been shown to be expressed at high levels at portals of pathogen entry, including the lung [34, 35]. Dectin-1 is a major *β*-glucan receptor on leukocytes [36] and can mediate a variety of cellular responses including endocytosis, phagocytosis, the respiratory burst, DC maturation, and the induction of cytokine/chemokine production [28, 29]. Like the TLRs, Dectin-1 can directly induce the development of adaptive immunity, particularly Th17 and Th1 responses [20, 37].

Increasing data from *in vivo* studies, although not entirely consistent, have demonstrated an important role for Dectin-1 in antifungal immunity [38, 39]. This receptor has been shown to mediate recognition of several fungal pathogens and to play an essential role in mouse models of infection with *Candida, Aspergillus,* and* Pneumocystis* [40–43]. In humans, deficiencies in Dectin-1 and in CARD9 have recently been linked to susceptibility to fungal infections [19, 44]. 

Dectin-1 signalling is mediated by the ITAM-like motif also known as hemITAM. The ITAM motifs typically contain an amino sequence comprising of a duplicate YxxL/I motif (YxxL/Ix_6−12_YxxL/I), where Y is tyrosine, L is Leucine, I is Isoleucine, and x denotes any amino acid [45, 46]. Upon ligand engagement, the ITAM-like motif of Dectin-1 becomes tyrosine phosphorylated by Src kinases, providing a docking site for Syk kinase, which links the Dectin-1 signalling to NF-*κ*B activation via the CARD9-Bcl10-MALT1 complex [17, 18]. In addition to the Syk-pathway Dectin-1 can signal via a second pathway mediated by the serine-threonine kinase Raf-1, which integrates with the Syk pathway at the point of NF-*κ*B activation [37]. Furthermore, Dectin-1 can also trigger the activation of NFAT, which regulates the induction of early growth response (Egr) family transcription factors, Egr-2 and Egr-3 [47]. This activation has been shown to occur through a pathway that requires phospholipase C-*γ*2 (PLC-*γ*2) [48]. Signalling from Dectin-1 also interacts with pathways induced by other receptors, such as the TLRs, resulting in the synergistic induction of cytokines including TNF, IL-10, and IL-23 [49–51].

## 5. Dectin-1 Recognition of Mycobacteria

Although the involvement of Dectin-1 in antifungal immunity has been extensively characterized [29], the role of this receptor in the control of mycobacterial infections remains poorly understood. Studies from a number of laboratories have implicated the role of Dectin-1 in response to mycobacterial infections, although the ligand involved is still unknown. 

**Figure 1:**
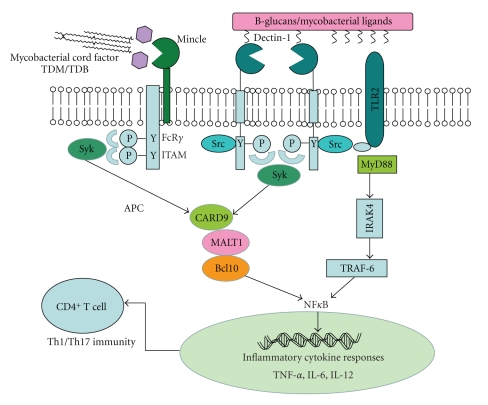
Mincle- and Dectin-1-mediated signalling in response to mycobacteria. Upon recognition of their mycobacterial ligands, Dectin-1 and Mincle signal via the Syk/CARD9 pathway for the production of inflammatory cytokines and the induction of Th1 and Th17 responses. There is also evidence that Dectin-1 signalling can cooperate with TLR2 to induce protective immunity.

In the initial study, Yadav and Schorey demonstrated that blocking Dectin-1 function with monoclonal antibodies inhibited the production of TNF-*α* by bone-marrow-derived murine macrophages (BMMø) infected with *M. smegmatis* [52]. Cytokine production was also impaired in the TLR2-deficient macrophages, indicating the requirement of this toll-like receptor for the Dectin-1-facilitated proinflammatory responses. Dectin-1 was also shown to be required for the production of RANTES, IL-6, and G-CSF in BMMø infected with nonpathogenic strains such as attenuated *M. bovis* and avirulent *M. tuberculosis* H37Ra (see Figure 1). Interestingly, Dectin-1 was not required for the induction of macrophage proinflammatory responses upon infection with virulent *M. tuberculosis* strain, H37Rv [52]. The authors argued that the minimal macrophage proinflammatory response induced by the virulent mycobacterium might be due to limited engagement of Dectin-1 by this strain [52]. However, in other studies (see below) Dectin-1 was found to interact with virulent mycobacteria. 

Rothfuchs et al. subsequently showed that Dectin-1 is important for IL-12p40 production in murine splenic dendritic cells (SpDCs) infected with MTB [53]. The authors demonstrated that blockade of Dectin-1 with laminarin resulted in the reduction of IL-12p40 production by these cells. In addition, the SpDCs from Dectin-1^−/−^chimeric mice displayed reduced levels of IL-12p40 when compared to the cells from the WT chimeras following stimulation with live MTB or *M. bovis*. Furthermore, the production of IL-12p40 by the MTB-stimulated WT SpDCs was impaired by the inhibition of Syk kinase with piceatannol [53]. However, in these experiments TLR2 deficiency had no effect. Rothfuchs et al. further showed that a soluble Dectin-1-Fc fusion protein [54] could bind to *M. bovis* BCG in a laminarin-inhibitable manner [53]. 

Using the nontuberculous mycobacterium, *M. abscessus*, Shin et al. demonstrated that the interaction of Dectin-1 and TLR2 is required for the efficient murine macrophage uptake of this organism and subsequent Syk activation leading to the production of TNF-*α*, IL-12p40, and IL-6 [55] (see Figure 1). The observations on TLR2-Dectin-1 cooperation are in agreement with the work by Yadav and Schorey [52], but differ with that of Rothfuchs et al. [53]; however, it should be noted that each of these studies focussed on different cell types and mycobacterial species and/or strains. Shin et al. also demonstrated the importance of Dectin-1/Syk pathway in the production of ROS by BMMø infected with *M. abscessus* [55]. 

More recently, the role of Dectin-1 in MTB-induced innate immune responses has been examined in human cells. Lee et al. have shown that the expression of Dectin-1 mRNA and protein in A549 airway epithelial cells was MTB-inducible in a TLR2-dependent manner. These authors also demonstrated the role of Dectin-1 in the production of ROS, proinflammatory cytokines, and antimicrobial activity during the intracellular growth of MTB in these cells [56]. 

In another study, Van de Veerdonk et al. showed that Dectin-1 and TLR4 are the key receptors for the induction of *M. tuberculosis*-triggered IL-17A responses in human PBMCs [57]. Furthermore, these authors demonstrated that the blockade of IL-1*β* signalling with IL-1RA resulted in the significant decrease of IL-17A production by the PBMCs infected with MTB. van de Veerdonk et al. concluded that the endogenous IL-1 pathway plays a central role in the induction of the MTB-induced Th17 response in human PBMCs [57]. 

Using human monocyte-derived DCs, Zenaro et al. have shown that stimulation of these cells with MTB leads to the Dectin-1-dependent production of TNF-*α*, IL-6, IL-1*β*, and IL-23, enabling DCs to “instruct” CD4^+^ lymphocytes to produce IL-17 and IFN-*γ*. The authors further investigated the effect of other receptors on these Dectin-1-dependent responses and demonstrated that stimulation of the DCs with biglycan and ManLAM (ligands of DC-SIGN and MR, resp.) increased MTB-triggered T-cell production of IFN-*γ* but resulted in the reduced amounts of IL-17. The authors concluded that Dectin-1 interaction with MTB-stimulated DCs induces Th1 and Th17 responses and that the DC-SIGN and MR engagement with DCs blocks the Dectin-1-triggered mechanisms, therefore inhibiting Th17 and favouring Th1 responses [58]. 

To explore the role of this receptor in response to *M. tuberculosis in vivo,* we characterized the effect of Dectin-1-deficiency in mice following aerosol infection with *M. tuberculosis* H37Rv (MJM and GDB, paper in press). We found a significant and reproducible reduction (~0.5 log) in the pulmonary bacilli burdens in the Dectin-1-deficient mice compared to the wild-type animals, but this did not correlate with any changes in pulmonary pathology, cytokine production, and ability to resist infection. Thus these data suggest that Dectin-1 plays a minor role in antimycobacterial immunity* in vivo*, at least in murine models. 

More work is still required to understand the exact role of this receptor in antimycobacterial immunity, and in particular, the mycobacterial ligand(s) recognised by this receptor. Mycobacteria do not possess *β*-glucans, so it is likely that Dectin-1 recognises a novel ligand on these pathogens. The role of Dectin-1 in human mycobacterial infections also warrants further investigation.

## 6. Mincle: An ITAM-Coupled FcR*γ*-Dependent Signalling Receptor

Mincle is a type II C-type lectin belonging to the “Dectin-2 cluster” of receptors [59, 60]. Also known as Clec4e and Clecsf9, Mincle was first identified by Matsumoto et al. as a protein whose expression was induced by lipopolysaccharide (LPS) [61]. The gene expression of Mincle was also shown to be induced in peritoneal macrophages by several proinflammatory cytokines, including IFN-*γ*, TNF-*α*, and IL-6 [61]. However, Mincle expression was demonstrated to be severely impaired in response to these stimuli in the NF-IL-6-deficient macrophages indicating that Mincle is a transcriptional target for NF-IL-6 [61]. Like other receptors in the Dectin-2 cluster, particularly Dectin-2 and DCAR, Mincle is expressed predominantly on cells of myeloid lineage, including dendritic cells and macrophages [59, 61]. This receptor is also expressed on B cells as well as on microglia in the brain [59, 62]. Structurally, Mincle contains a single extracellular carbohydrate recognition domain, a transmembrane domain, and a short cytoplasmic domain without a known signalling motif [61]. However, typical of the Dectin-2 cluster of receptors, Mincle possesses a positively charged residue near the transmembrane region through which it associates with the adaptor FcR*γ* to trigger intracellular signalling via Syk/CARD9 pathway [63, 64]. A report by Yamasaki et al. has demonstrated that cross-linking Mincle on the surface of peritoneal macrophages resulted in the production of proinflammatory cytokines which were abrogated in FcR*γ*-deficient cells as well as CARD9-deficient cells [64]. 

Mincle can act as a receptor for both endogenous and exogenous ligands, having been shown to recognise PAMPs from a number of fungal pathogens, including *C. albicans, Saccharomyces cerevisiae, *and *Malassezia *species [65–67]. Specifically, Wells et al. reported that Mincle recognises *C. albicans* and that although this receptor did not act as a phagocytic receptor, it played a role in TNF*α* production in RAW264.7 and BMMøs in response to this fungus. Furthermore, they demonstrated that Mincle-deficient mice displayed a significantly greater fungal burden in the kidneys compared to wild-type mice during systemic candidiasis [66]. Bugarcic et al. further demonstrated that recombinant CRDs of both human and mouse Mincle could recognise *C. albicans *[65].

In contrast, Yamasaki et al. reported that *C. albicans *was not recognised by Mincle using a cell-based reporter system, but rather that the receptor specifically recognised *Malassezia* species (a pathogen associated with skin diseases such as tinea versicolor, folliculitis, and atopic dermatitis) [67]. However, the strains of Candida screened were different from those reported by Wells et al. [66], and Yamasaki and coworkers have suggested that Mincle may distinguish between different strains of *C. albicans *[67]. Recognition of *Malassezia *by Mincle was shown to require the mannose binding EPN-motif in the receptor's CRD, as well as Ca^2+^, and *α*-mannose was identified as the ligand of this receptor [67]. Stimulation of BMMø with *Malassezia* resulted in the production of MIP-2, KC, IL-10, and TNF-*α*, which was significantly reduced in the Mincle-deficient macrophages [67]. Additionally, intraperitoneal injection of this fungus resulted in impaired IL-6 and TNF*α* production and neutrophil infiltration in Mincle-deficient mice compared to wild-type mice [67].

In addition to the role in fungal recognition, Yamasaki et al. have demonstrated that Mincle senses necrosis. Using an NFAT-GFP-based reporter system the authors found that GFP expression was greatly increased in the presence of necrotic cells, as well as with supernatants from necrotic cells and lysates generated from normal cells. Mutation of the EPN motif in the CRD did not reduce recognition of the dead cells, suggesting that Mincle recognised the endogenous ligand in a carbohydrate independent manner. The authors subsequently identified spliceosome-associated protein 130 (SAP130), a preformed soluble factor released upon necrosis, as a ligand for this receptor. Stimulation of peritoneal macrophages or Mincle expressing T cell hybridomas with SAP130 resulted in MIP-2 and IL-2 production, respectively. *In vivo*, the authors demonstrated that inflammatory responses induced by this PRR promoted the infiltration of neutrophils to the site of necrosis, induced either by whole body irradiation or by peritoneal injection of necrotic cells [64]. 

## 7. Mincle-Mediated Immunity to Mycobacteria

In addition to fungi, Mincle can recognise MTB, an activity which requires the EPN motif within the carbohydrate recognition domain of this receptor [63]. To identify the specific ligand for this receptor in mycobacteria, Ishikawa et al. analyzed various components of *M. smegmatis *lipid extracts using chromatographic techniques. These screening experiments revealed TDM as the mycobacterial ligand for Mincle. TDM is made up of a trehalose moiety and two mycolate chains [68], components which could not individually activate Mincle-expressing cells, indicating that a combination of both the sugar and the lipid moieties of this glycolipid are important for its interaction with Mincle [63]. Another independent report by Schoenen et al. has confirmed Mincle as the major receptor of TDM [69]. 

As discussed above, the mycobacterial cord factor TDM and its synthetic analogue TDB can activate the Syk-CARD9-Bcl10 pathway and induce antimycobacterial immunity [25]. Mincle was identified as the main receptor initiating this signalling pathway and driving the TDM/TDB-induced antimycobacterial immunity [63, 69]. Loss of Mincle resulted in greatly reduced production of inflammatory cytokines and nitric oxide *in vitro *[63, 69]. Further *in vitro* analysis also demonstrated that the TDM/TDB-induced expression of G-CSF and IL-1*β* in BMMø was abrogated in the FcR*γ* ^−/−^ cells, confirming the involvement of the FcR*γ* adaptor chain in Mincle-mediated signalling [69]. *In vivo*, Mincle-deficient and FcR*γ*-deficient mice had decreased TDM-induced IL-6 and TNF production in the sera [69]. These results indicate the critical roles of Mincle and FcR*γ* in the innate immune responses induced by the mycobacterial glycolipids. 

To investigate the role of Mincle in driving TDB-induced cellular immunity, Schoenen et al. analysed T cell responses in the draining lymph nodes of wild-type and Mincle-deficient mice following vaccination with the subunit vaccine H1 (an MTB fusion protein of Ag85B and ESAT-6). Compared to the wild-type mice, which produced robust amounts of IFN-*γ* and IL-17, there were significantly reduced T cell numbers in the lymph nodes of Mincle-deficient mice when TDB was used as an adjuvant, indicating that Mincle is responsible for the Th1 and Th17 responses induced by this glycolipid [69]. The T cell responses were also FcR*γ* dependent [69]. Thus, the Mincle-FcR*γ* signalling pathway is crucial in the induction of Th1 and Th17 protective immunity that is triggered by the mycobacterial glycolipids in this setting (see Figure 1). Collectively, these data suggest that Mincle is a key receptor involved in the induction of the CARD9 signalling pathway in response to MTB.

## 8. SIGNR3 in Host Response to *M. tuberculosis* Infection

Other Syk-dependent receptors have been implicated in the control of *M. tuberculosis *infection. Recently a study by Tanne et al. reported that SIGNR3, a murine homologue of DC-SIGN, that signals via intracellular hemITAM is important for early immune response to *M. tuberculosis* infection [70]. The authors demonstrated that SIGNR3 can directly interact with live* M. tuberculosis* and promote the ability of fibroblasts to mediate endocytosis and to take up ManLAM [70]. To investigate the effects of SIGNR3 deficiency on host resistance to mycobacterial infection, the authors infected SIGNR3 knockout and wild-type mice with *M. tuberculosis* and compared their ability to control the infection. Although the SIGNR3-deficient mice had significantly higher amounts of the lung bacilli, their ability to mount Th1, Th2, Tc1, and Th17 responses was similar to the wild-type mice. In addition, there were no differences in the formation of granulomatous lesions and the survival patterns between the SIGNR3-deficient and the control mice [70]. These results indicated that SIGNR3 is important in early host resistance to *M. tuberculosis* but redundant for the establishment of long-term defence against the infection [70].

Tanne et al. also demonstrated that stimulation of the SIGNR3-expressing macrophages with either ManLAM or live *M. tuberculosis *resulted in the production of high amounts of IL-6 and TNF in comparison with the control cells [70]. Specific inhibition of Syk by piceatannol abolished SIGNR3 signalling and the cytokine production in a dose dependent manner, allowing the authors to conclude that the SIGNR3-mediated production of IL-6 and TNF occurred in a Syk-dependent manner [70]. Conclusively, the work by Tanne et al. provided evidence that the Syk-coupled CLR SIGNR3 plays a role in early host resistance to *M. tuberculosis* in mice [70]. 

Because of the differences between SIGNR3 and the related human molecule, DC-SIGN, it is difficult to speculate on the implication of the current murine SIGNR3 data in the human infections. DC-SIGN utilizes a different signalling mechanism from the mouse homologue; it does not require Syk kinase for signalling [71], but its interaction with ManLAM leads to the activation of serine/threonine kinase Raf-1 [72]. *In vitro* work has shown that DC-SIGN can be exploited by *M. tuberculosis* to evade the host immune surveillance [73]. Furthermore, a clinical study investigating effects of polymorphisms in the gene of DC-SIGN has suggested that decreased level of DC-SIGN is associated with increased protection against TB [74].

## 9. Concluding Remarks

Many PRRs that recognise mycobacteria have been identified, but those studied to date appear to be largely redundant when examined *in vivo*. The identification of the Mincle and the CARD9 pathway provides the first evidence for an essential nonredundant signalling that is crucial for antimycobacterial immunity. However, more work is needed to determine if Mincle promotes protective or nonprotective responses during Mtb infection *in vivo*. Furthermore, given the number of PRRs that can activate the Syk/CARD9 pathway it is likely that there are other receptors feeding into this pathway. Special attention should be given to FcR*γ*-associated PRRs, such as Dectin-2 which also recognise MTB [75]. Identifying these receptors, and understanding their roles during infection, will be critical if we wish to use this information for the development of novel adjuvants and/or vaccines.
